# Aortic Valve Reconstruction with Ozaki Technique

**DOI:** 10.21470/1678-9741-2020-0476

**Published:** 2022

**Authors:** Gabriella Ricciardi, Raoul Biondi, Gabriele Tamagnini, Mauro Del Giglio

**Affiliations:** 1 Department of Cardiothoracic Surgery, Leiden University Medical Center, Leiden, Netherlands.; 2 Department of Cardiac Surgery, Villa Torri Hospital, GVM Care & Research, Bologna, Italy.

**Keywords:** Aged, Bioprosthesis, Aortic Valve, Heart Valve Prosthesis, Aorta, Heart Valve Prosthesis Implantation

## Abstract

Modern bioprostheses offer a complete and definitive solution to elderly patients who need aortic valve surgery. Nonetheless, the scenario is more demanding when dealing with younger and less fragile patients. In this setting, any prosthetic aortic valve replacement can provide only a suboptimal solution and its related issues have not been fixed yet. The answer to the needs of this special population is the enhancement and refinement of the surgical technique. The Ozaki technique relies on custom-tailored autologous aortic cusps individually sutured in the aortic position. This approach has been showing optimal results if performed after a dedicated training period.

**Table:** 

Abbreviations, acronyms & symbols	
AM	= Anterior mitral leaflet
v	= Atrial side
AVNeo	= Aortic valve neocuspidization
LA	= Left atrium
LCA	= Left coronary artery
LCC	= Left coronary cusp
NCC	= Non-coronary cusp
RA	= Right atrium
RCA	= Right coronary artery
RCC	= Right coronary cusp
STJ	= Sinotubular junction
VS	= Ventricular side

## INTRODUCTION

The treatment of aortic valve diseases is one of the oldest surgical challenges Cardiac Surgery still must face. Since the implantation of the first Starr-Edwards caged-ball prosthesis in 1960, the evolution and progress of the construction and the design of prosthetic valves led to the biological revolution first, and, currently, to the transcatheter era. If modern biological solutions offer a complete and definitive path to those elderly patients who need aortic valve surgery, the scenario is more demanding when we deal with younger and less fragile patients.

Durability of both surgical bioprosthesis and percutaneous valves is a well-known issue in this population and the burden of either reintervention or patient-prosthesis mismatch following a percutaneous valve-in-valve procedure must be considered when a 50-year-old or younger patient suffers from severe aortic valve disease.

In this scenario, a prosthetic aortic valve replacement can provide only a suboptimal solution and the industrial technology has not fixed its related issues yet.

The answer to the needs of this special population is the enhancement and refinement of the surgical technique. To date, only two procedures can avoid the drawbacks of a long-term anticoagulation or the burden of one or multiple reinterventions, namely the Ross operation and the aortic valve neocuspidization (AVNeo, with the Ozaki technique). Both require expert hands, appropriate training, and optimization of the surgical technique.

The use of autologous pericardium in cardiac surgery started in the 1960s, when Bjoerk and Hultquist first implanted autologous pericardial leaflets. Subsequently, in 1986, Love et al. reported the immersion of autologous pericardium in 0.6% glutaraldehyde for 10 minutes to eliminate the problems related to its scarring. After that report, Al Halees published a series of glutaraldehyde-fixed autologous pericardial valves on 65 young patients. Nonetheless, all these approaches failed for the lack of reproducibility and the poor longevity of patch material when implanted in younger patients.

The method developed by Ozaki et al. relies on custom-tailored autologous aortic cusps individually sutured in the aortic position.

## TECHNIQUE

The Ozaki technique for aortic valve reconstruction ^[1,2]^ is based on the independent replacement of the three aortic valve cusps with tailored autologous pericardial neocusps. The preparation and the tailoring of the patient’s pericardium is therefore one of the cornerstones of this procedure. Thus, it is the first aspect we will put on focus in the following description.

In order to achieve enough tissue for the three cusps, a minimum amount of 7×7 cm of pericardium cleaned from fat and redundant tissue of the outer surface need to be excised. However, autologous fresh pericardium could present some relevant problems. Its elastic and twisty properties make handling the patch potentially difficult. In addition, when left untreated, it exhibits a high propensity to develop fibrosis and calcification. Hence, to address this pitfall, the autologous patch is currently and routinely treated with a 0.6% glutaraldehyde solution for 10 minutes and then rinsed three times with saline solution for a total of 20 minutes.

**Fig. 1:**
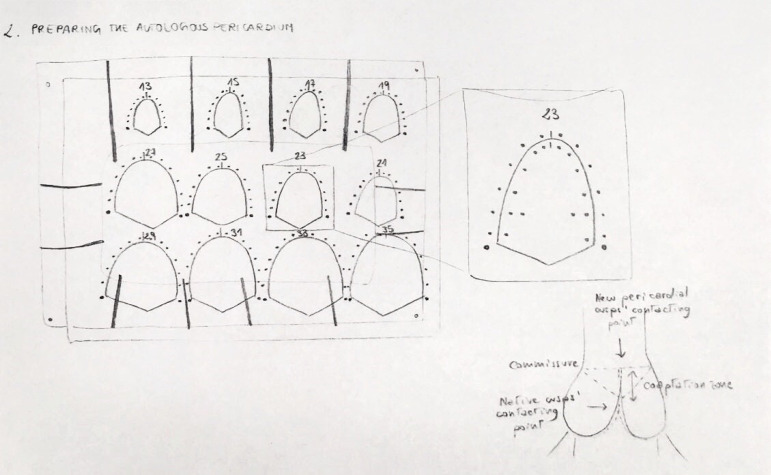
Dedicated Ozaki sizer. AM=anterior mitral leaflet; AS=atrial side; LA=left atrium; LCC=left coronary cusp; NCC=non-coronary cusp; RA=right atrium; RCC=right coronary cusp; STJ=sinotubular junction; VS=ventricular side

**Fig. 2:**
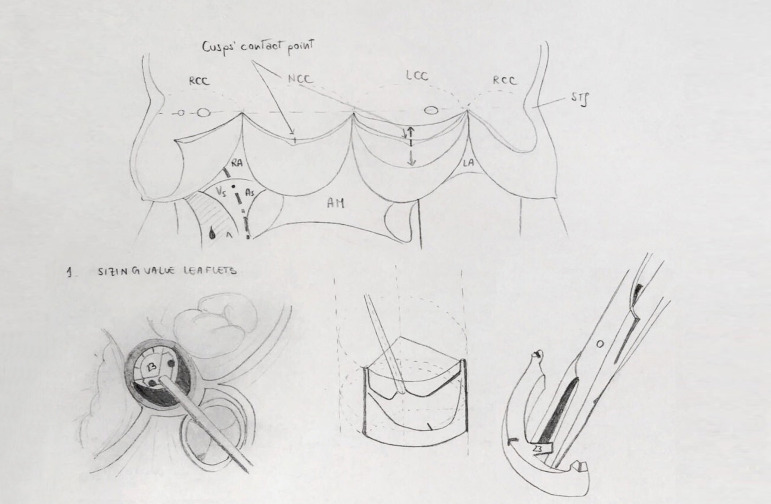
Pericardial template.

Once the aortic valve is exposed and the diseased cusps are excised, an extensive and accurate annular decalcification is crucial. It is paramount to achieve a precise measurement of the cusps after this step. A dedicated Ozaki sizer (Figure 1) is used to measure the distance between each commissure. The autologous pericardium is then tailored with the original Ozaki template (Figure 2). During the trimming procedure careful attention should be paid to use the thinner part of the pericardial patch for the reconstruction of the smaller cusp, in order to improve its mobility, while the thicker part should be reserved for the biggest, so the tolerance to diastolic stress can be ensured.

**Fig. 3:**
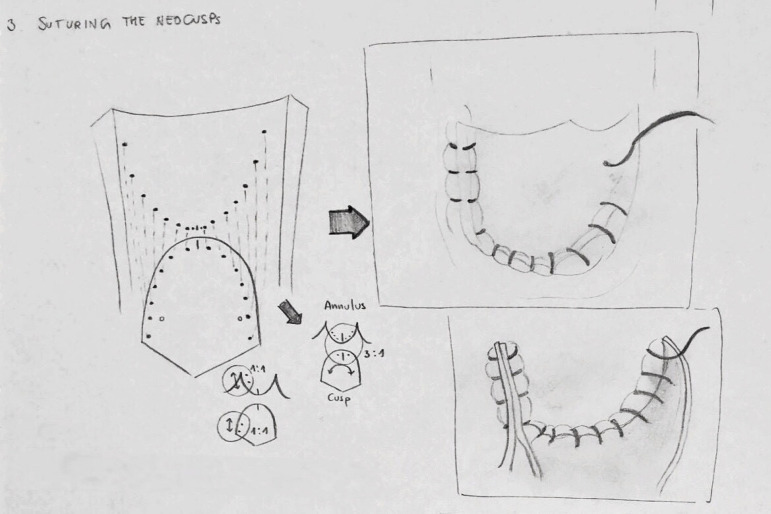
Stitch distribution and annular-neocusp ratio.

**Fig. 4:**
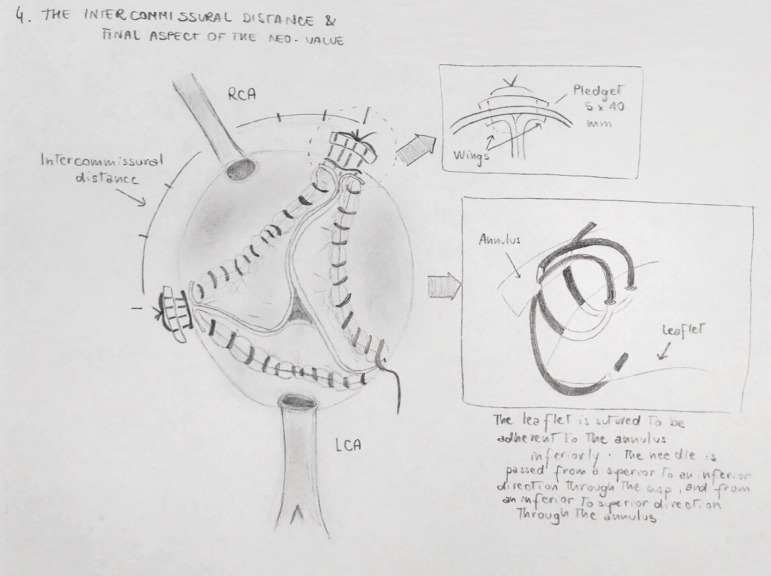
Intercommissural distance and final aspect of the neovalve. LCA=left coronary artery; RCA=right coronary artery

The final part of the reconstruction involves suturing the neocusp to the aortic annulus (Figure 3), usually using a single running 4-0 monofilament stitch. The suture starts at the nadir of the annulus, where the monofilament is tied down. The ends of the suture are then used to move the reconstruction bidirectionally towards the commissures. It is important during this step to place the cusp with its inner side facing the left ventricular surface. Another critical aspect of the reconstruction at this point entails the correct spacing of the bites: the distance between each bite on the autologous pericardium must be regular and fixed, while on the aortic annulus it must be different at the nadir and the commissural zone. As the suture comes closer to the nadir, the distance between the bites on the aortic annulus gets shorter than the distance on the cuspid, with a 3:1 ratio (Figure 3). Moving towards the commissure, this discrepancy normalizes itself, with a perfect correspondence between the bites of the running suture at the level of the coaptation zone. In this area, the lateral margin of each cusp is sewed with a little plication of the inner pericardium facing the aortic wall. This fashion warrants the cusp’s maximized resistance to the stress, and, at the same time, optimizes the coaptation drawing the pericardium towards the other cusps. An additional commissuroplasty 4-0 monofilament stitch is placed through the neocusps, above the last bite of the running suture, and then laterally on the edge of the cusp plication, with the aim of securing the coaptation point and fitting the plicated cusp to the aortic wall. Both the running annular suture and commissural stitch are tied on the outward side of the aorta, with the interposition of a felted pledget (Figure 4). After the resuspension of the three neocusps, a visual check - enhanced with negative pressure made by the left ventricular vent - is needed to evaluate the degree of the coaptation.

## DISCUSSION

The reconstruction of the aortic cusps based on the technique described by Ozaki et al. ^[1,2]^ is highly reproducible and shows some peculiar and unique features. Since the pericardium is sewed directly to the aortic annulus, this operation offers a maximized valve orifice area with physiologic transvalvular gradients. At the same time, the absence of a stented frame preserves the aortic root physiological ability to expand its diameter during the systolic ejection, thus maintaining the natural coordination between left ventricle, aortic annulus, sinus of Valsalva, and ascending aorta ^[3]^. In addition, resuspending the three neocusps up to the level of the sinotubular junction provides an extensive coaptation area during diastole. The extent of this zone, also known as effective height, is one of the most powerful predictors of the long-term valve competence, as already suggested in the literature ^[4,5]^. The large coaptation obtained with the AVNeo operation, combined with the previously exposed features, is indeed one of the reasons for the excellent mid- and long-term results demonstrated in the series by Ozaki et al. and the literature ^[6]^, which are the basis of the hypothetical prolonged durability of this surgical reconstruction.

Unlike what it might be thought, this technique is feasible also when managing bicuspid or unicuspid aortic valves, providing their “tricuspidalization”. Tricuspidalization of a bicuspid or unicuspid valve with the Ozaki techniques ensures the possibility to restore the typical orientation of the cusps of a normal valve and the achievement of an appropriate length of the free margin of leaflets, thus permitting its fully opening while maintaining the normal valve shape. In an unicuspid valve, the total length of the free edge of the leaflets is significantly shorter compared to a tricuspid valve. Furthermore, aortic valve reconstruction can be adopted not only for aortic regurgitation but also in case of valve stenosis, infective endocarditis, and reoperation after bioprosthesis deterioration.

## CONCLUSION

Surgical valve replacement represents the gold standard to treat aortic valve diseases, but the landscape of possibilities in this field is growing. The need to improve and enhance the performance of the surgical offer is mandatory, particularly in those patients who get otherwise convicted to a suboptimal treatment. Different drawbacks can be acknowledged either in mechanical or bioprosthetic solutions. Despite the need for lifelong anticoagulation, the choice of a mechanical prosthesis is the recommended option for patients under 60 years, even if thromboembolic events, device malfunction, and spontaneous bleeding in the late decades are considerable disadvantages that usually concern and blur the patient’s choice. Similarly, concerns are driven by the limited durability of the bioprostheses in young patients. Surely, the presence of small aortic annuli, the young age at surgery, and the pediatric population represent a cluster in which there is a lack of durable and tested solutions. In a recent work by Del Nido et. al. ^[6]^, the Ozaki procedure also demonstrated promising results in children. Particularly, in patients with small annuli undergoing aortic root enlargements and valve reconstruction, the native annuli continued to grow appropriately and remained free from subsequent aortic stenosis. Thus, the refinement of the AVneo operation could represent a more suitable yet versatile option.

In addition, it is also our belief that this procedure can be mastered after a relatively short training period, considering the high reproducibility and ease of all the steps required to properly perform it thanks to the significant standardization of the original surgical technique.

**Table:** 

Authors' roles & responsibilities	
GR	Substantial contributions to the conception of the work; drafting the work; final approval of the version to be published
RB	Substantial contributions to the conception of the work; drafting the work; final approval of the version to be published
GT	Substantial contributions to the conception of the work; drafting the work; final approval of the version to be published
MDG	Substantial contributions to the conception of the work; drafting the work; final approval of the version to be published
